# 

**Published:** 1886-05-20

**Authors:** 


### EDITORIAL NOTICES.

CORRECTIONS.—In our April number is a translation on "Enuresis Nocturna" which should have been credited to the Southern California Practitioner, an excellent journal published at Los Angelos, Cal.

In same number, in review of that able work entitled "A System of Practical Medicine," by William Pepper, M.D., LL. D., the name "Prof. Agnew D. Hays" should have been Prof. D. Hays Agnew, and "Prof. Harrison Allen" should have been Prof. Allen Harrison, and "Prof. S. S. Davis" should have been Prof. N. S. Davis.

C. P. FRICK, the gentlemanly agent of the house of R. A. Robinson & Co., Louisville, Ky., called and presented samples of their beautiful preparations, Wine of Coca and Syrup of Hypophosphites. The formulæ of these preparations are given, and they are elegantly put up.

MEDICAL AND SURGICAL HISTORY OF THE WAR.—The last volume of this work, by Dr. Charles Stewart, Surgeon in the U. S. Navy, is completed. It contains 900 pages, and is well illustrated.

KAVA KAVA.—We are in receipt of a lecture on *Piper Menthysticum*, Kava Kava. Sent out by Parke, Davis & Co. The lecture was delivered by Dr. L. Lewin, of the University of Berlin, and gives something of the nature and properties of this drug. The plant is found in New Guinea and other tropical regions. It is used by the natives as a nerve excitant, and may be classed with like agents in other countries, which possess properties to cause agreeable sensations, or a certain degree of intoxication. It is now being introduced into this country; its proper uses and therapeutical effects are not yet fully understood.

RECEIPTED.

For 1885—Drs. V. T. Lindsay, C. A. Jordan, J. R. Green, J. R. Johnson, Thomas Marks, Wm. Wirt, J. A. King, A. J Davis, F. C. Davis, G. W. Bowling, J. E. Carter.

For 1886—A. J. Talbot, J. W. Baker, J. H. Duggan, to July; C. M. Gibson, G. B. Battle, C. B. Thomas, J. B. Wright, to March, 1884; William Ramsey.

